# Identification of Amino Acids Essential for Viral Replication in the HCMV Helicase-Primase Complex

**DOI:** 10.3389/fmicb.2018.02483

**Published:** 2018-10-23

**Authors:** Gaetan Ligat, Sandra Da Re, Sophie Alain, Sébastien Hantz

**Affiliations:** ^1^U1092, RESINFIT, CHU Limoges, INSERM, University of Limoges, Limoges, France; ^2^CHU Limoges, Laboratoire de Bactériologie-Virologie-Hygiène, National Reference Center for Herpesviruses, Limoges, France

**Keywords:** human cytomegalovirus, helicase-primase, molecular modeling, mutagenesis, antiviral drugs

## Abstract

Promising new inhibitors that target the viral helicase-primase complex have been reported to block replication of herpes simplex and varicella-zoster viruses, but they have no activity against human cytomegalovirus (HCMV), another herpesvirus. The HCMV helicase-primase complex (pUL105-pUL102-pUL70) is essential for viral DNA replication and could thus be a relevant antiviral target. The roles of the individual subunits composing this complex remain to be defined. By using sequence alignment of herpesviruses homologs, we identified conserved amino acids in the putative pUL105 ATP binding site and in the putative pUL70 zinc finger pattern. Mutational analysis of several of these amino acids both in pUL105 and pUL70, proved that they are crucial for viral replication. We also constructed, by homology modeling, a theoretical structure of the pUL105 N-terminal domain which indicates that the mutated conserved amino acids in this domain could be involved in ATP hydrolysis.

## Introduction

Human cytomegalovirus (HCMV), a beta herpesvirus, infects 50–90% of the population worldwide. Although it is mostly symptomless in immunocompetent individuals, HCMV infection is a major cause of morbidity and mortality in transplant recipients (Torres-Madriz and Boucher, 2008) and other immunocompromised individuals. Globally, HCMV represents the first viral cause of birth defect, leading to severe congenital malformations (Leruez-Ville and Ville, 2017). Approved HCMV inhibitors (ganciclovir, cidofovir, and foscarnet), target the viral DNA polymerase pUL54. These drugs suffer from poor oral bioavailability, tolerability and cannot be used during pregnancy. Furthermore, prolonged prophylactic treatment selects for resistance mutations in viral polymerase pUL54 and/or the kinase pUL97 (viral kinase needed for phosphorylation of the HCMV inhibitor ganciclovir) (Alain et al., 2004; Hantz et al., 2010; Lurain and Chou, 2010; Andouard et al., 2016). New drugs targeting essential viral proteins other than pUL54 are therefore urgently needed. Phase III clinical trials include two such drugs, only in transplant setting: letermovir targets the HCMV terminase pUL56 (Lischka et al., 2010; Goldner et al., 2011; Melendez and Razonable, 2015) while maribavir targets the kinase pUL97 (Biron et al., 2002; Alain et al., 2013). In addition, two helicase-primase inhibitors have also been developed against alpha-herpesviruses: pritelivir (previously named BAY 57-1293 or AIC316), a thiazolylamide active against herpes simplex virus types 1 and 2 (HSV-1 and -2) (Wald et al., 2014), and amenamevir (or ASP2151), an oxadiazolylphenyl derivative, active against both HSV and varicella-zoster virus (VZV or HHV-3) (Chono et al., 2010). They have been proved as efficient as acyclovir: IC50 of 0.02 μM against HSV1-2 for pritelivir and 0.03 μM for amenamevir with a CC50 > 30 μM for both drugs. However, their precise site of action has not been elucidated to date and these drugs are not active against the beta-herpesviruses like HCMV (Dropulic and Cohen, 2010).

**FIGURE 1:**
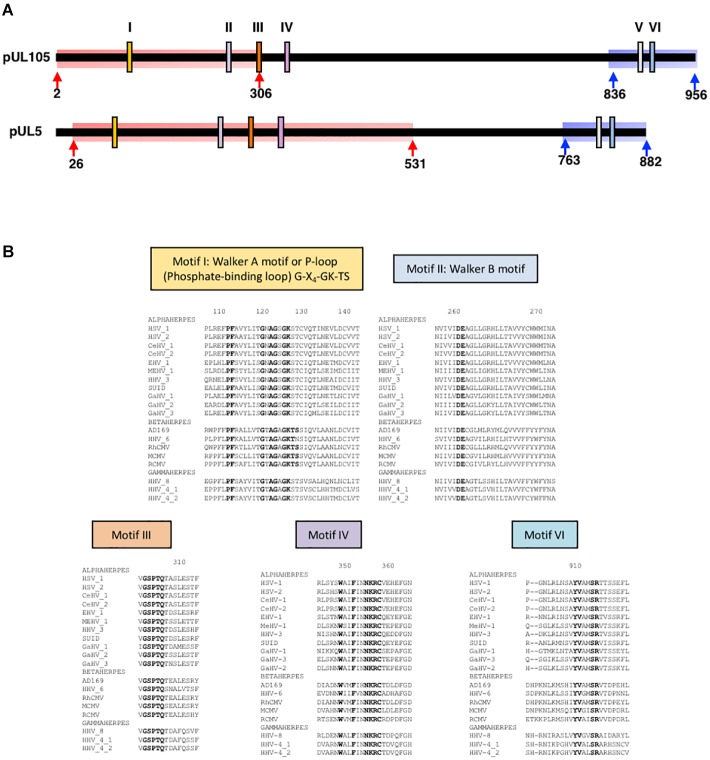
Conserved regions and amino acids in herpesviruses helicases. **(A)** Schematic structure of HCMV helicase subunit pUL105 and HSV-1 helicase subunit pUL5 with conserved motifs I to VI (according to Fairman-Williams et al., 2010) as indicated with their position at scale along each protein. Arrows delimitate highlighted regions for which a structural homology model could be built for pUL105 and pUL5 (Red: N-terminal domain model; Blue: C-terminal domain model); the number of the first and last residues taken into account in the structural homology models are indicated. **(B)** Sequences alignments of conserved regions of pUL105 (motifs I, II, III, IV, and VI) with homologs from 18 herpesviruses belonging to alpha, beta, and gamma sub-families of herpesviruses as indicated. Sequence numbering is consistent with residues of the HCMV reference strain AD169. Conserved key residues are shown in bold letters.

In HCMV, the herpesvirus helicase-primase complex is composed of a helicase (pUL105), a primase (pUL70) and a primase-associated factor (pUL102) (McMahon and Anders, 2002). These ORFs were first identified as essential for oriLyt-dependent DNA replication (Pari and Anders, 1993). Conserved regions and putative functional patterns have been identified in the proteins forming the HCMV helicase-primase complex (Woon et al., 2008). The primase pUL70 contains several conserved motifs or regions, including a region (residues 881–920) that corresponds to a putative zinc-finger pattern shown to play essential role in HSV-1 (Biswas and Weller, 1999). The helicase pUL105 contains six motifs (I–VI) typical of the superfamily 1 of helicase proteins (SF1 helicases) (Fairman-Williams et al., 2010; Figure 1A), motifs I and II corresponding, respectively, to the Walker A (or P-loop) and Walker B motifs in helicase proteins (Walker et al., 1982). The Walker A motif, present in many nucleotide binding proteins, such as helicases, binds ATP and forms a phosphate binding loop (Saraste et al., 1990; Story and Steitz, 1992). The Walker B motif contains highly conserved residues and when in complex with ADP or ATP, the first conserved D residue in this motif is in close proximity to the ATPase A motif and binds an Mg^2+^ ion with a water molecule (Pai et al., 1990; Story and Steitz, 1992). The SF1 helicase includes three families: the UvrD-like/Rep family, Pif-1-like family, and Upf1-like family (Fairman-Williams et al., 2010). Structural data at various ATP-hydrolysis states have been described for helicases of the three families, such as the *Escherichia coli* helicase UvrD (Lee and Yang, 2006), the *Deinococcus radiodurans* helicase RecD2 (Pif-1-like family) (Saikrishnan et al., 2008) and the human RNA helicase Upf1 (Cheng et al., 2007).

The fact that amenamevir and pritelivir are not active against HCMV (Dropulic and Cohen, 2010) indicates potential structural differences in the helicase-primase complex between alpha and beta herpesviruses. For better characterization of the structure-function relationships of the HCMV helicase-primase complex, we first compared the protein sequences of 18 herpesviruses homologs of pUL105 and pUL70. Then, we built up a theoretical structure of the pUL105 N-terminal fragment, based on the helicase domain of the human RNA helicase Upf1 (Cheng et al., 2007). Finally, we used a mutational approach to investigate the importance of amino acids potentially involved in ATP binding (pUL105) or zinc chelation (pUL70). This allowed identification of amino acids in the helicase-primase complex that are crucial for viral replication. Differences in the structural models of the HCMV and HSV-1 helicases that could explain specificity of antiviral drugs action were also highlighted.

## Results

### Determination of pUL105 Theoretical Structure and Identification of Amino Acids Likely to Be Involved in ATP Binding

Comparing HCMV clinical strains, Woon et al. (2008) identified six conserved regions (motifs I–VI) in the *UL105* sequence (Figure 1A). To identify amino acids that might be involved in the catalytic activity of the HCMV helicase, we aligned pUL105 sequence to 18 herpesviruses homologs sequences of different origins (11 alpha, 4 beta, and 3 gamma herpesviruses; Supplementary Table S1). By focusing on pUL105 regions potentially involved in ATP binding (Walker et al., 1982), we identified several highly conserved amino acids, namely P112, F113, G120, A122, G123, G125, and K126 in motif I for all herpesviruses classes, plus T127 and S128 for beta-herpesviruses, as well as D261 and E262 in motif II, and G302, S303, P304, T305, and Q306 in motif III (Figure 1B). Our results confirmed and completed the previous alignment made on five herpesviruses motifs I and II (Martignetti and Barrell, 1991).

**FIGURE 2:**
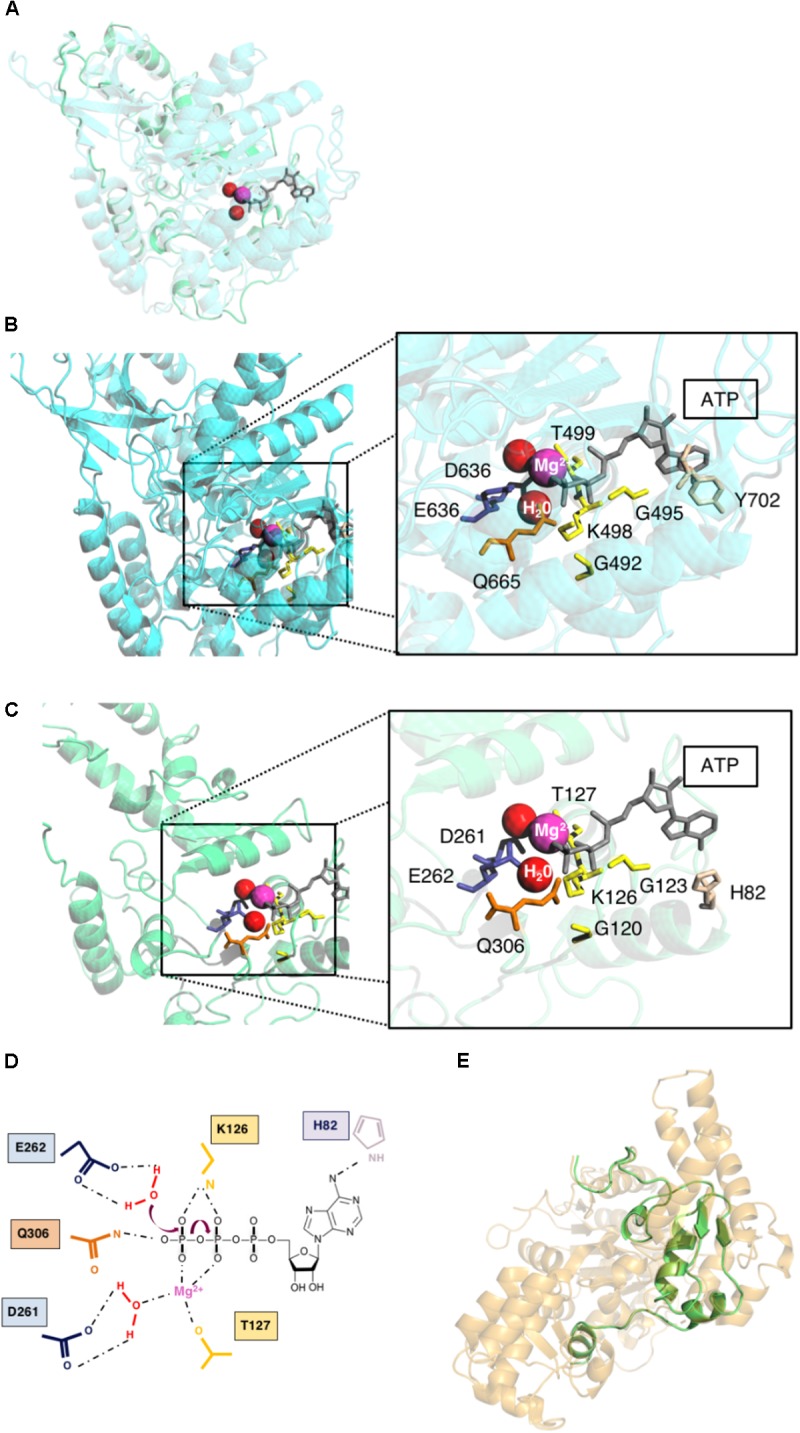
Theoretical structure of pUL105. **(A)** Superposition of homology modeling of pUL105 (2–306) in green and human Upf1 helicase template in blue with ATP (gray), water molecules (red) and Mg^2+^ ion (magenta). **(B)** Human Upf1 helicase with bound ATP (gray), water molecules (red), and Mg^2+^ ion (magenta). Magnification of Upf1 ATP binding site with highlighting of amino acids in close proximity to Mg^2+^ ion, water molecules and ATP. **(C)** pUL105 (2–306) theoretical structure and magnification of the putative ATP binding site highlighting amino acids in close proximity to Mg^2+^ ion, water molecules and ATP. **(D)** Schematic drawing of the putative ATPase reaction and amino acids involved in the helicase pUL105 active site. **(E)** Superposition of homology modeling of pUL105(867–950) in green and *E. coli* RecD helicase template in orange.

In order to determine which of these amino acids might be involved in ATP binding, we attempted to build a first homology model of pUL105 using CPHmodels-3.2 server. While using the whole pUL105 sequence, we obtained a homology model for the pUL105 N-terminal domain (residues 2–306, encompassing motifs I, II, and III) only (score 4.6). To refine the model, we made the query with the N-terminal part of pUL105 (residue 1–309) and obtained a model for pUL105 (2–306) (score 5.9). Models were based on the coordinates of the crystal structure of the human Upf1 helicase domain bound to the ATP analog AMPPNP (Cheng et al., 2007) used as template by the CPHmodels-3.2 server. As shown in Figure 2A, the theoretical structure of pUL105 (2–306) superimposed neatly on the known Upf1 helicase domain structure.

Cheng et al. (2007) have experimentally demonstrated that, amino acids D636, E637 and Q665 in Upf1 are only involved in ATPase activity, while K498, R703, and R865 are involved in both ATP binding and hydrolysis. These authors suggested that SF1 helicases share a similar ATP binding and hydrolysis mechanism. ATP, water molecule and Mg^2^+ were thus inserted in the theoretical structure of the putative active domain of pUL105 (2–306), as in the Upf1 helicase domain bound to AMPPNP. It clearly appeared that several of the conserved amino acids identified above (G120, G123, K126, T127, D261, E262, and Q306) could potentially belong to the pUL105 ATP binding site (compare Figures 2B,C). Thus, our structural model of pUL105 suggests that K126, D261, E262, and Q306 could be involved in ATP binding and/or ATP hydrolysis. Amino acids Y702 and R703 in domain motif IV and amino acid R865 in motif VI of the Upf1 helicase are also involved in ATP binding and hydrolysis (Cheng et al., 2007) and are highly conserved in SF1 helicases. Amino acids R357 and R915, in pUL105 motifs IV and VI, respectively, are conserved among herpesviruses and could be structurally equivalent to R703 and R865 of Upf1. While there seem to be no equivalent to Upf1 Y702 in pUL105 motif IV (a conserved K instead among herpesviruses; Figure 1B), the theoretical structure of pUL105 (2–306) shows the presence of a histidine residue at position 82 that could potentially be involved in ATP binding (Figures 2C,D).

As motifs V and VI are conserved among SF1 helicases, we attempted to build a homology model of the C-terminal part of pUL105 encompassing both domains. We obtained a homology model of pUL105 (867–950) C-terminal domain (84 residues) based on the coordinates of the crystal structure of the RecD helicase in the *E. coli* RecBCD:DNA complex, used as template by the CPHmodels-3.2 server (Singleton et al., 2004) (score: 7.2; Figure 2E). The RecD subunit is an helicase with 5′-3′ directionality belonging to the Pif-1 like family of SF1 helicase, and as observed for pUL105, the usually conserved Y residue of motif IV is absent in RecD motif IV (Fairman-Williams et al., 2010).

### Several Amino Acids in the pUL105 Putative ATP Binding Site Are Essential for Viral Replication

To investigate the contribution of the conserved amino acids to viral replication, we produced recombinant EGFP-expressing HCMV-BAC viruses with point mutations in pUL105 (Table 1). The mutations were chosen such as to change the side-chain length or the functional group, in order to determine whether the size or charge is important for the function. Therefore, in motif I, glycine 120 and 123 were replaced by a serine or a valine (G120S, G120V, G123S, and G123V), lysine 126 was replaced by an alanine or an asparagine (K126A and K126N), and threonine 127 was replaced by an alanine (T127A). In motif II, aspartate 261 was replaced by a glutamate or an asparagine (D261E and D261N) and glutamate E262 was replaced by an aspartate or a glutamine (E262D and E262Q). Glutamine 306, in motif III, was replaced by an alanine (Q306A). In addition, to determine if histidine 82 might belong to the catalytic site (see above), H82 was replaced by a lysine or an arginine (H82K and H82R).

**Table 1:** Impact of HCMV-BAC-*UL105* mutants in putative pUL105 ATP-binding site on growth in cell culture (fibroblasts MRC-5).

HCMV-BAC	Day 4 post-transfection	Day 11 post-transfection
*UL105* WT	-	++
H82K	-	+
H82R	-	+
G120S	-	-
G120V	-	-
G123S	-	-
G123V	-	-
K126A	-	-
K126N	-	-
T127A	-	-
D261E	-	-
D261N	-	-
E262D	-	-
E262Q	-	-
Q306A	-	-

*-, absence of viral growth; ++, foci*.

**FIGURE 3:**
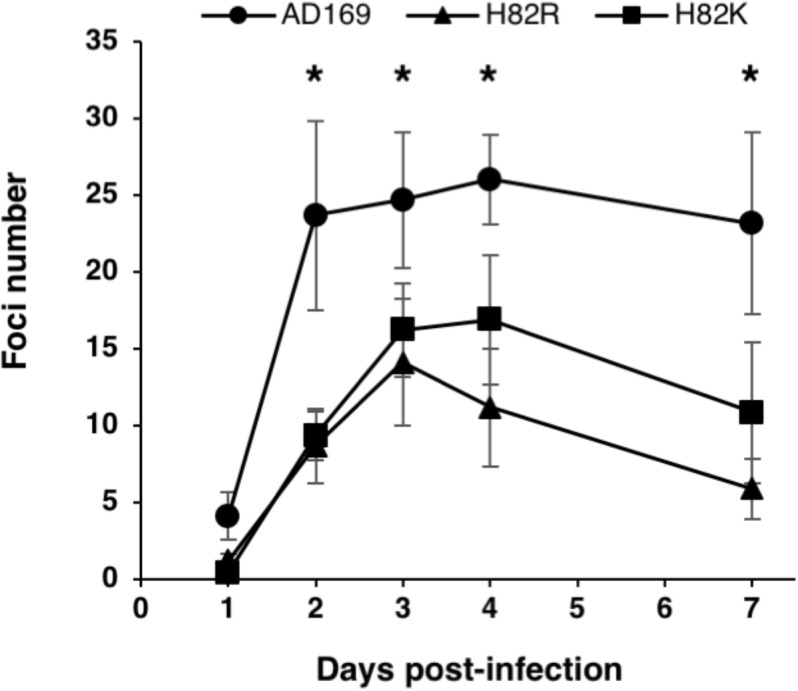
Effect of the helicase pUL105 H82 mutations on viral growth. Growth curves of the virus parental strain HCMV-BAC AD169 and its recombinant strains HCMV-BAC *UL105* H82K and *UL105* H82R. Fluorescent foci were counted daily from day 1 to day 7. Data are the average of three independent experiments. ^∗^*p* < 0.05 (Mann–Whitney test).

The HCMV-BAC mutants and the wild-type HCMV-BAC were transfected into MRC-5 human fibroblasts, and viral replication was monitored for 11 days. Among mutants, only H82K and H82R produced infectious foci (Table 1 and Supplementary Figure S1), indicating that mutations of amino acids G120, G123, K126, T127, D261, E262, or Q306 drastically impaired viral replication and propagation in cell culture. Contrary to the wild-type HCMV-BAC, which formed large foci on day 11, only small foci were obtained with the H82K and H82R mutants (Table 1 and Supplementary Figure S1). To estimate the fitness impact of these mutations on virus replicative capacity, we then compared the growth curves of the wildtype and mutant viruses. Both mutants grew more slowly than the wildtype virus (Figure 3).

Altogether, these results demonstrate that residues G120, G123, K126, T127, D261, E262, and Q306, are crucial for HCMV replication.

### Comparison of HCMV pUL105 and HSV pUL5 Helicases

**FIGURE 4:**
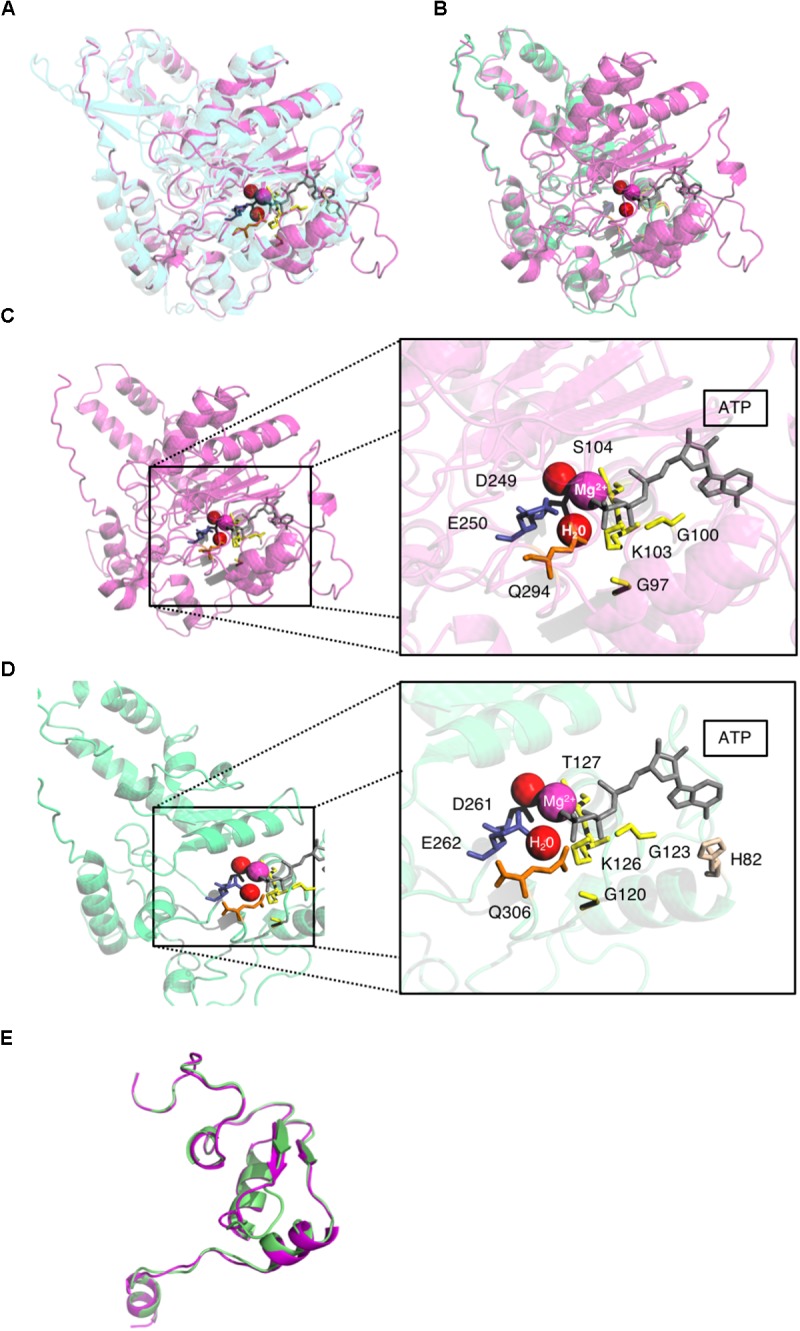
pUL5 and pUL105 share the same theoretical structure. **(A)** Superposition of homology modeling of pUL5 (26–531) in magenta and human Upf1 helicase template in blue with ATP (gray), water molecules (red), and Mg^2+^ ion (magenta). **(B)** Superposition of theoretical structures of pUL5 (26–531) in magenta and pUL105 (2–306) in green with ATP (gray), water molecules (red), and Mg^2+^ ion (magenta). **(C)** pUL5 (26–531) theoretical structure with magnification of the putative ATP binding site highlighting amino acids in close proximity to Mg^2+^ ion, water molecules and ATP. **(D)** pUL105 (2–306) theoretical structure with magnification of the putative ATP binding site highlighting amino acids in close proximity to Mg^2+^ ion, water molecules and ATP. **(E)** Superposition of homology modeling of pUL105 (867–950) in green and pUL5(795-876) in magenta.

All six conserved helicase motifs of the HSV-1 helicase pUL5 have also proved to be essential for HSV DNA replication and several of the conserved amino acids (G102, D249, and E250) to be involved in ATP-binding/hydrolysis (Zhu and Weller, 1992; Graves-Woodward et al., 1997). These findings support the hypothesis that the homologous residues identified in pUL105 could also be involved in ATP binding and/or hydrolysis. To assess this potential similarity of function at the structural level, we also built a first homology model of the HSV-1 helicase. The homology models of the N- and C-terminal portions of pUL5 were built based on the templates used for pUL105. The pUL5 N-terminal part, pUL5 (20–531), was built based on the Upf1 helicase domain bound to the ATP analog AMPPNP crystal structure (Cheng et al., 2007) (score: 6.2; Figure 4A). The pUL5 C-terminal part, pUL5 (795–876), was built based on the RecD helicase crystal structure (score: 7.5; Figure 4E). As expected from the models, the theoretical structures of pUL5 (26–531) and pUL105 (2–306) superimposed neatly on each other (Figure 4B). G120, G123, K126, D261, E262, and Q306 amino acids in pUL105 (Figure 4D) are structurally equivalent to G97, G100, K103, D249, E250, and Q294 in pUL5 (Figure 4C). This strong similarity comforts the hypothesis that D261 and E262 in pUL105 are involved in ATP-binding/hydrolysis as shown for D249 and E250 in pUL5 (Graves-Woodward et al., 1997). Compared to pUL105, a larger portion of the HSV-1 pUL5 helicase N-terminal domain superimposed with the human Upf1 helicase domain (compare Figures 2A, 4A). Considering the C-terminal domain, homology models of HCMV and HSV-1 helicases were comparable in length (respectively, 84 and 82 amino acids) and structure (Figure 4E).

### The Putative Zinc Finger Pattern of pUL70 Is Required for Viral Replication

**FIGURE 5:**
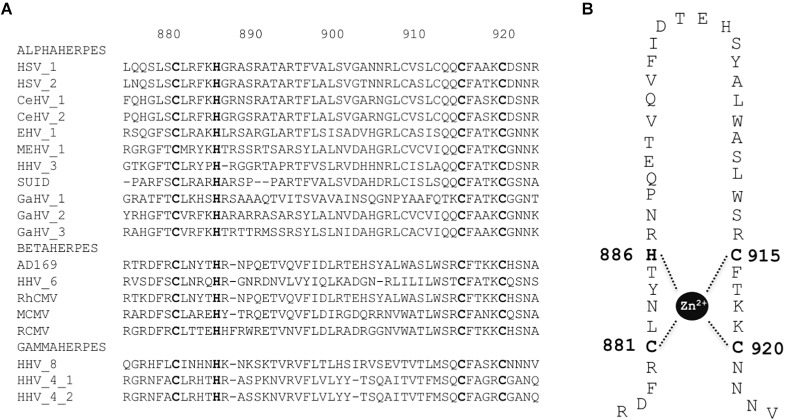
Conserved zinc finger region and amino acids in primase pUL70. **(A)** Sequences alignment of the zinc finger conserved region of pUL70 with homologs from 18 herpesviruses belonging to alpha, beta, and gamma sub-families of herpesviruses as indicated. Sequence numbering is consistent with residues of the HCMV reference strain AD169. Conserved key residues involved in the formation of the zinc-finger motif are shown in bold letters. **(B)** Representation of the putative zinc finger motif: CX_2_CX_22_CXH (CCCH zinc finger). Amino acids involved in zinc ion binding are numbered.

The primase subunit of the helicase-primase complex (pUL70) displays a putative DNA binding pattern with a putative zinc finger motif (Woon et al., 2008). We aligned the sequences of HCMV pUL70 homologs from the 20 herpesviruses used above (Supplementary Table S2). Within the putative metal-binding pattern of pUL70, we identified three cysteines (C881, C915, and C920) and one histidine (H886) that are highly conserved among herpesviruses (Figure 5A) and that could be directly involved in zinc ion binding (Figure 5B).

To investigate the role of these residues in viral replication, we produced recombinant EGFP- expressing HCMV-BAC viruses (C881S, H886A, C915S, and C920S) and tested their ability to replicate in MRC-5 cells. Unlike wildtype HCMV-BAC, none of the mutants formed infectious foci after 11 days of culture (Table 2 and Supplementary Figure S2), indicating that these mutations drastically impair viral replication and propagation in cell culture.

**Table 2:** Impact of HCMV-BAC-*UL70* mutants in putative pUL70 zinc-finger pattern on growth in cell culture (fibroblasts MRC-5).

HCMV-BAC	Day 4 post-transfection	Day 11 post-transfection
*UL70* WT	-	++
C881S	-	-
H886A	-	-
C915S	-	-
C920S	-	-

*-, absence of viral growth; ++, foci*.

Amino acids C881, H886, C915, and C920 from cysteine-rich metal-binding motif in the C-terminal domain of HCMV pUL70 are thus essential for viral replication and could form the zinc finger motif C881-X_4_-H886-X_28_-C915-X_4_-C920 (CHCC).

## Discussion

The helicase-primase of HCMV is essential for viral replication and thus represents a potential target for the development of new anti CMV compounds. However, no crystal structure is available to date and structure-function relationships are not clearly defined. Using BAC analysis, our functional studies showed that conserved amino acids in pUL70 zinc finger domain (C881, C915, C920, and H886) and in pUL105 domains involved in ATP binding and hydrolysis (G120, G123, K126, D261, E262, and Q306), are essential for viral replication and propagation (Tables 1, 2). Our results on pUL70 are in agreement with previous results on HSV1 primase pUL52, showing that conserved cysteines of pUL70 zinc finger region are essential for DNA binding and primase activities of pUL52 (Biswas and Weller, 1999). pUL52 zinc finger region was also proposed as to be involved in the optimal binding of the HSV1 pUL5 helicase and its ATPase and helicase activities (Biswas and Weller, 1999). We propose that the potential metal-binding motif involving the cysteine and histidine residues within the sequence C881-X_4_-H886-X_28_-C915-X_4_-C920 is essential for proper pUL70 folding and might be required for DNA binding. Interestingly, a single mutation, A899T, conferring resistance to pritelivir, has been located near the zinc-finger domain of the pUL52 primase in HSV-1 (Field and Biswas, 2011), suggesting that this region might be one of the potential target for pritelivir.

K126, D261, E262, and Q306 amino acids in pUL105 are structurally equivalent to amino acids shown to be involved in ATPase activity and/or ATP binding and hydrolysis in HSV1 pUL5 [D249 and E250 (Graves-Woodward et al., 1997)] and human Upf1 helicases [K498, D636, E337, and Q665 (Cheng et al., 2007)]. Our study proved that they are essential for viral replication and propagation. There is now a need for more studies focusing on a better characterization of the role of the identified conserved amino acids at the level of the enzymatic activity of pUL105 and pUL70.

By taking advantage of the existence of tridimensional structures of SF1 family helicases, we could build theoretical models of the HCMV pUL105 and HSV1 pUL5 helicases. The homology model for both proteins was built through CPHmodels-3.2 server that used as template the coordinates of the crystal structure of the human Upf1 helicase domain bound to the ATP analog AMPPNP (Cheng et al., 2007). These models showed that the theoretical structures of pUL5 (26–531), pUL105 (2–306) and the helicase domain of Upf1 superimposed neatly on each other. They showed that conserved amino acids in Walker A and Walker B motifs (G120, G123, K126, T127 and D261, E262 respectively in pUL105 (2–236), and homologous amino acids in pUL5) (26–531), respectively, form the catalytic domain, by binding the β and γ phosphates of ATP through a coordinated Mg^2+^ and two water molecules to facilitate the nucleophile attack of ATP, as previously found in the ATPase center (Sun et al., 2007). These models are strongly comforted by our functional results and others (Graves-Woodward et al., 1997). Besides the similarities in the catalytic sites of the helicases, our theoretical homology models also highlighted some differences that could be important in differentiating the specificity of these helicases. Indeed, the highly conserved residue Y702 in Upf1 motif IV involved in ATP binding seems to have no equivalent in herpesviruses (Figure 1B). Instead, a H82 residue in pUL105 is in a close enough proximity to ATP molecule to potentially replace Y702 in ATP binding process (Figures 2C,D). Interestingly enough, alignment of herpesviruses helicase proteins sequences showed that the region (39 residues) containing this amino acid in pUL105 is missing in pUL5 and other herpesviruses (Supplementary Figure S3). As commonly observed in SF1 helicases (Fairman-Williams et al., 2010), both pUL105 and pUL5 contain an insert between motifs IV and V compared to Upf1, pUL105 insert being 70 bp longer than that of pUL5. The presence of these insert might actually explain why, when trying to obtain a theoretical structure of the whole proteins, only the N-terminal portion of the proteins could be modeled. Interestingly, a larger portion of pUL5 than pUL105 matched Upf1 structure. The homology model for pUL105 encompassed residues 2–306, i.e., 304 amino acids containing conserved motifs I to III (Figure 1A). The homology model for pUL5 contained 500 amino acids (residues 26–531) including protein sequence beyond motif IV (Figure 1A). These observations indicate that the structure of the HCMV and HSV-1 helicase are very similar for their N-terminal part up to motif III and may differ afterward, which suggests potential differences in the structure-function relationship between HCMV and other herpesviruses. Surprisingly enough, mutations in the pUL5 helicase conferring HSV-1 resistance to pritelivir or amenamevir, such as N342K, G352V, M355T, K356Q, or K356N, all map to a small region located within motif IV and just downstream (Field and Biswas, 2011). These results strongly suggest that these two drugs may target the region around this motif. Considering the location of these resistances and the potential structural difference after motif III between pUL5 and pUL105, as revealed by our homology models, we can assume that this structural difference might explain why pritelivir or amenamevir are only active against the alpha-herpesviruses HSV and VZV and not against the beta-herpesvirus HCMV.

Our work comfort previous studies in highlighting the essential role, for HCMV viral replication, of conserved residues in homologous domains between HCMV pUL70 and HSV-1 pUL52 primase proteins (potential zinc finger involved in interaction of the helicase-primase complex with DNA) as well as between herpesviruses helicases pUL105 (HCMV) and pUL5 (HSV-1) and human helicase Upf1. Nevertheless, the residues identified in this study are surely not the only ones to be important, and other residues (conserved or not among herpesviruses) might also be indispensable for HCMV replication. The first modeling step of HCMV and HSV-1 helicases presented here allowed to give essential indications about the potential structure-function relationships of these proteins. It highlighted similarities and differences within herpesviruses helicases that might explain specificity of anti-viral drugs against alpha-herpesviruses. Although these models need to be confirmed by molecular dynamics experiments, and more functional studies are required to confirm these hypotheses, they are a first step for a better understanding of the structure-function relationships of viral helicase-primase complex indispensable for the development of new anti-HCMV drugs.

## Materials and Methods

### Sequence Alignment and Homology Modeling

The pUL105 and pUL70 amino acid sequences of HCMV reference strain AD169 (Chee et al., 1990) were aligned with the sequences of 19 homologous pUL105 and pUL70 proteins from other herpesviruses (described in Supplementary Tables S1, S2, respectively). Alignments were performed with the Clustal Omega (Ω) multiple sequence alignment (MSA) tool provided by the EMBL-EBI bioinformatics web and programmatic tools framework (Li et al., 2015). The CPHmodels-3.2 (Nielsen et al., 2010) server was used to build the theoretical structure of (i) the N-terminal fragment of pUL105(2–306) and pUL5(26–531), using the human Upf1 helicase domain bound to AMPPNP as template (PDB: 2GJK) (Cheng et al., 2007); (ii) the C-terminal fragment of pUL105 (867–950) and pUL5 (795–876), using the *E. coli* RecBCD-DNA complex as template (PDB: 1W36) (Singleton et al., 2004).

### Human and Bacterial Cells

MRC-5 human fibroblasts (bioMérieux, France) were cultured at 37°C with 5% CO_2_ in minimal essential medium (MEM) containing 10% fetal bovine serum and antibiotics. *E. coli* strain GS1783 was used for BAC mutagenesis (Borst et al., 1999). HCMV-BAC contains the EGFP (enhanced green fluorescent protein) gene in the unique short region of the HCMV genome and was derived from parental strain pHB5, the BAC-cloned genome of the HCMV laboratory strain AD169 (Chee et al., 1990; Borst et al., 1999).

### BAC Mutagenesis

Amino acid substitutions in pUL105 and pUL70 were made by *en passant* mutagenesis, using a two-step markerless Red-recombination system for BAC mutagenesis in *E. coli* strain GS1783. *UL105* and *UL70* point mutations were introduced into an EGFP-expressing HCMV-BAC (Chee et al., 1990; Borst et al., 1999) yielding several mutants. The primers used for mutagenesis are described in Supplementary Table S3. The presence of mutations in the *UL105* and *UL70* genes of each virus was confirmed by sequencing prior to transfection. The primers used for sequencing are described in Supplementary Table S4. We have previously shown that *en passant* mutagenesis does not introduce other mutations that could have a negative impact on viral replication (Ligat et al., 2017).

### Reconstitution of Mutant Viruses

The impact of the mutations on viral growth was assessed by transfecting mutated HCMV-BAC into MRC-5 fibroblasts (bioMérieux, France) using the Transfast^TM^ liposomal reagent (Promega, United States) as recommended by the manufacturer.

### Plaque Assays and Growth Curve Analysis

To estimate the impact of each mutation on viral fitness, we inoculated viral recombinant strains and AD169-EGFP in 48-wells MRC-5 cell cultures with a multiplicity of infection (MOI) of 0.01. From day 1 to day 7 post-inoculation, the number of fluorescent cytopathic foci was counted to establish viral growth curves for each recombinant. The curves displayed represent the average of three independent experiments. The Mann-Whitney test was used for statistical analysis. ^∗^*p* < 0.05.

## Author Contributions

GL designed and performed the research experiments, analyzed the statistical, wrote the manuscript, and prepared the figures. SD edited the manuscript. SA and SH coordinated the research and manuscript writing. All authors reviewed the manuscript.

## Conflict of Interest Statement

The authors declare that the research was conducted in the absence of any commercial or financial relationships that could be construed as a potential conflict of interest.
