# Supplementary material for: Identification of Amino Acids Essential for Viral Replication in the HCMV Helicase-Primase Complex
Source: Front Microbiol. 2018 Oct 23;9:2483. doi: 10.3389/fmicb.2018.02483 (PMC6205958; doi:10.3389/fmicb.2018.02483)
Supplement: Supplementary file 1 [file Table_1.docx]

Supplementary Materials for

**Identification of amino acids essential for viral replication in the HCMV helicase-primase complex**

Gaetan Ligat, Sandra Da Re, Sophie Alain & Sébastien Hantz

**Fig. S1: Plaque formation assay in MRC-5 cells after transfection of HCMV-BAC GFP *UL105* WT or mutated recombinant virus strains.** Formation of foci monitored at days 4 and 11 after transfection of MRC-5 cells with HCMV-BAC GFP *UL105* WT (AD169) or its mutated derivatives as indicated. Green fluorescent foci or single infected cells are shown by white and grey arrow respectively.

**Fig. S2: Plaque formation assay in MRC-5 cells after transfection of HCMV-BAC GFP *UL70* WT or mutated recombinant virus strains.** Formation of foci monitored at days 4 and 11 after transfection of MRC-5 cells with HCMV-BAC GFP *UL70* WT (AD169) or its mutated derivatives as indicated. Green fluorescent foci or single infected cells are shown by white and grey arrow respectively.

**Fig. S3:** **Sequences alignment of the region of pUL105 with homologues from 18 herpesviruses containing the H82 residue.** Sequence numbering is consistent with that of the HCMV reference strain AD169 residues.

**Fig. S1**


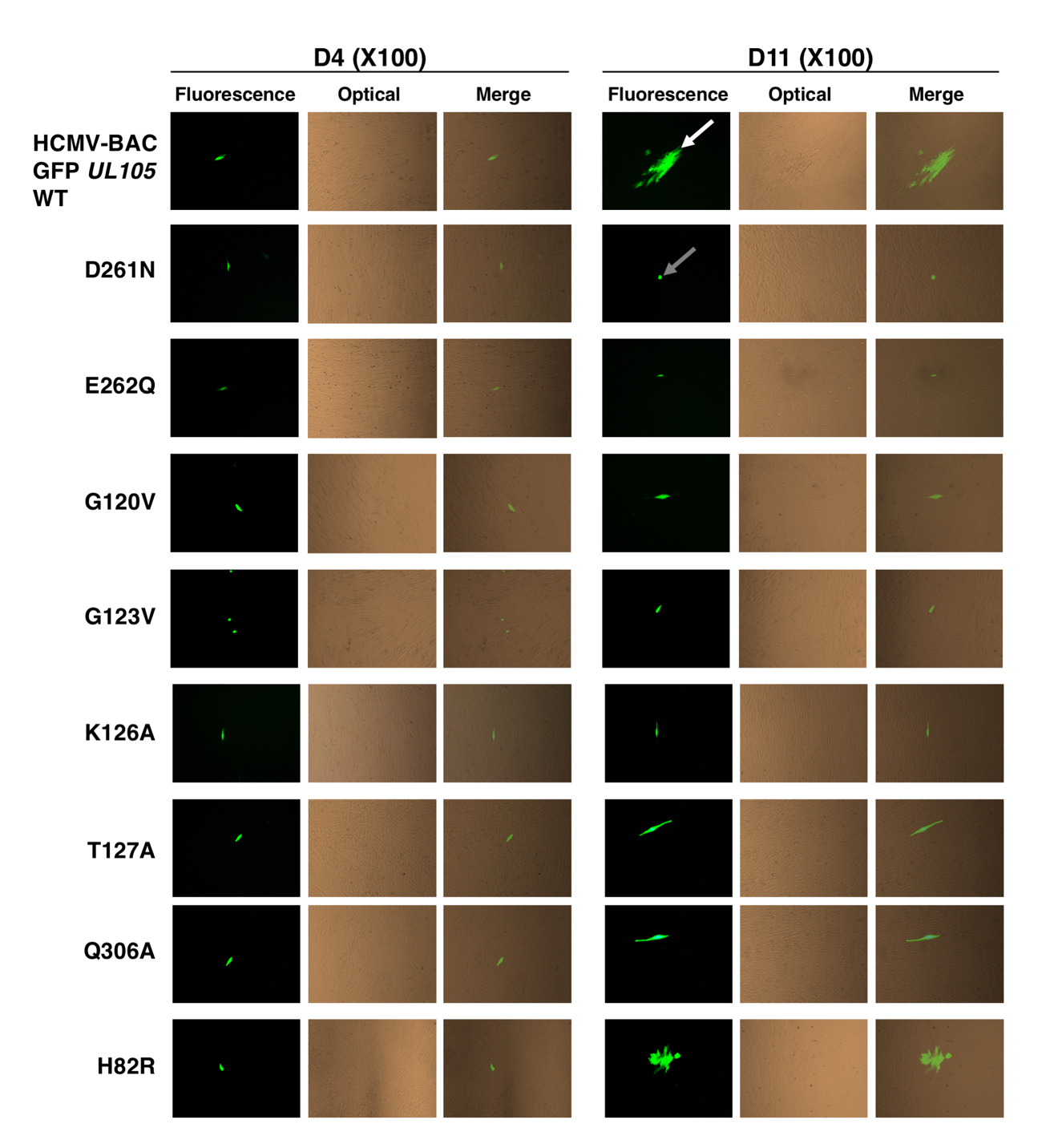


**Fig. S1: Plaque formation assay in MRC-5 cells after transfection of HCMV-BAC GFP *UL105* WT or mutated recombinant virus strains.** Formation of foci monitored at days 4 and 11 after transfection of MRC-5 cells with HCMV-BAC GFP *UL105* WT (AD169) or its mutated derivatives as indicated. Green fluorescent foci or single infected cells are shown by white and grey arrow respectively.

**Fig. S2**


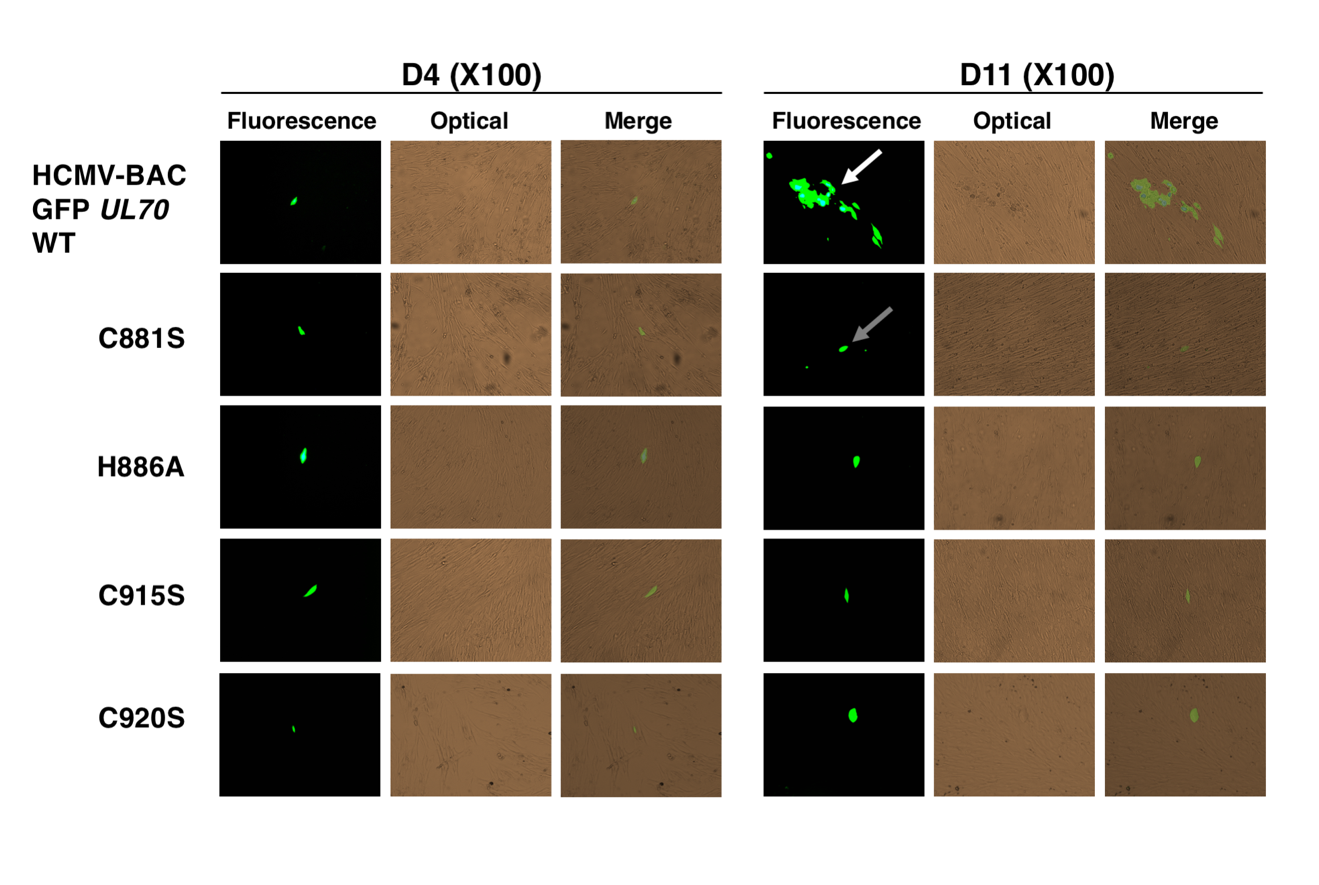


**Fig. S2: Plaque formation assay in MRC-5 cells after transfection of HCMV-BAC GFP *UL70* WT or mutated recombinant virus strains.** Formation of foci monitored at days 4 and 11 after transfection of MRC-5 cells with HCMV-BAC GFP *UL70* WT (AD169) or its mutated derivatives as indicated. Green fluorescent foci or single infected cells are shown by white and grey arrow respectively.

**Fig. S3**


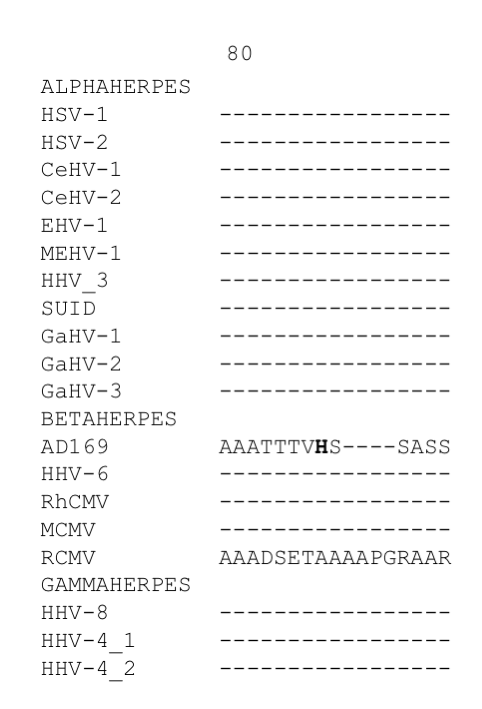


**Fig. S3:** **Sequences alignment of the region of pUL105 with homologues from 18 herpesviruses containing the H82 residue.** Sequence numbering is consistent with that of the HCMV reference strain AD169 residues. The H82 residue are shown in bold letter.

**Supplementary Table 1: Sequences used for alignment of pUL105 and homologues**

| **Name** | **Virus type** | **Herpesvirus**  **family** | **Accession number** | **Reference** |
| --- | --- | --- | --- | --- |
| HSV_1 | Human herpesvirus type 1 | alpha | AAA45819 | McGeoch et al., 1988 |
| HSV_2 | Human herpesvirus type 2 | alpha | AKN90872 | Collot et al., 2016 |
| CeHV_1 | Cercopithecine herpesvirus 1 | alpha | NP_851864 | Pelyrigina et al., 2003 |
| CeHV_2 | Cercopithecine herpesvirus 2 | alpha | YP_164447 | Tyler, Peters, and Severini, 2005 |
| EHV_1 | Equine herpesvirus 1 | alpha | YP_053101 | Telford et al., 1992 |
| GaHV_3 | Gallid herpesvirus 3 | alpha | NP_066835 | Izumiya et al., 2001 |
| MeHV_1 | Meleagrid herpesvirus 1 | alpha | NP_073298 | Afonso et al., 2001 |
| HHV_3 | Human herpesvirus 3 (Varicella-Zooster virus) | alpha | AFO85632 | Peters et al., 2012 |
| SUID | Suid herpesvirus 1 (Pseudorabies virus) | alpha | AAA50986 | Dean et al., 1994 |
| GaHV_1 | Gallid herpesvirus 1 | alpha | AER28164.1 | Lee,S.W., 2011 |
| GaHV_2 | Gallid herpesvirus 2 | alpha | YP_001033933 | Tulman et al., 2000 |
| AD169 | Human cytomegalovirus AD169 strain pUL105 | beta | P16736 | Chee et al., 1990 |
| HHV_6 | Human herpesvirus 6 | beta | APO37002 | Royle et al., 2016 |
| RhCMV | Cercopithecine herpesvirus 8 (Rhesus cytomegalovirus) | beta | AAZ80648 | Rivailler et al., 2006 |
| MCMV | Murid herpesvirus 1 (Murine cytomegalovirus) | beta | CAJ86580 | Sweet et al., 2007 |
| RCMV | Murid herpesvirus 2 (Rat cytomegalovirus Maastricht) | beta | AAF99194 | Vink et al., 2000 |
| HHV_8 | Human herpesvirus 8 | gamma | YP_001129396 | Rezaee et al., 2006 |
| HHV_4_1 | Human herpesvirus 4 type 1 | gamma | AGZ95191 | Tsai et al., 2013 |
| HHV_4_2 | Human herpesvirus 4 type 2 | gamma | YP_001129475 | Dolan A et al., 2006 |

| **Name** | **Virus type** | **Herpesvirus**  **family** | **Accession number** | **Reference** |
| --- | --- | --- | --- | --- |
| HSV_1 | Human herpesvirus type 1 | alpha | AFE62881 | Macdonald et al., 2012 |
| HSV_2 | Human herpesvirus type 2 | alpha | AEV91392 | NA |
| CeHV_1 | Cercopithecine herpesvirus 1 | alpha | NP_851913 | Pelyrigina et al., 2003 |
| CeHV_2 | Cercopithecine herpesvirus 2 | alpha | YP_164496 | Tyler, Peters, and Severini, 2005 |
| EHV_1 | Equine herpesvirus 1 | alpha | AII81101 | NA |
| GaHV_3 | Gallid herpesvirus 3 | alpha | NP_066885 | Izumiya et al., 2001 |
| MeHV_1 | Meleagrid herpesvirus 1 | alpha | NP_073347 | Afonso et al., 2001 |
| HHV_3 | Human herpesvirus 3 (Varicella-Zooster virus) | alpha | AFO85582 | Peters et al., 2012 |
| SUID | Suid herpesvirus 1 (Pseudorabies virus) | alpha | AJD79487 | NA |
| GaHV_1 | Gallid herpesvirus 1 | alpha | AER28112 | Lee et al., 2011 |
| GaHV_2 | Gallid herpesvirus 2 | alpha | YP_001033982 | Tulman et al., 2000 |
| AD169 | Human cytomegalovirus AD169 strain pUL70 | beta | ACL51139 | Bradley et al., 2009 |
| HHV_6 | Human herpesvirus 6 | beta | APO38512.1 | Royle et al., 2016 |
| RhCMV | Cercopithecine herpesvirus 8 (Rhesus cytomegalovirus) | beta | AAZ80600 | Rivailler et al., 2006 |
| MCMV | Murid herpesvirus 1 (Murine cytomegalovirus) | beta | CCE57397 | Smith et al., 2013 |
| RCMV | Murid herpesvirus 2 (Rat cytomegalovirus Maastricht) | beta | AAF99156 | Vink et al., 2000 |
| HHV_8 | Human herpesvirus 8 | gamma | ABD28907 | Rezaee et al., 2006 |
| HHV_4_1 | Human herpesvirus 4 type 1 | gamma | CEQ37212 | NA |
| HHV_4_2 | Human herpesvirus 4 type 2 | gamma | YP_001129457 | Dolan et al., 2006 |

**Supplementary Table 2: Sequences used for alignment of pUL70 and homologues.**

**Supplementary Table 3: Primers used for “en passant” BAC mutagenesis.**

| **BAC Mutants** | | **Primer sequence** | | |
| --- | --- | --- | --- | --- |
| *UL105* G120S-for | | CCGTTGGCCCTTCTTTCCCTTCCGCGCGCTGCTCGTCACCAGCACGGCGGGCGCCGGCAAGACTAGGGATAACAGGGTAATCGATTT | | |
| *UL105* G120S-rev | | CCAGCACCTGGATGCTGGAAGTCTTGCCGGCGCCCGCCGTGCTGGTGACGAGCAGCGCGCGGAGCCAGTGTTACAACCAATTAACC | | |
| *UL105* G120V-for | | CCGTTGGCCCTTCTTTCCCTTCCGCGCGCTGCTCGTCACCGTCACGGCGGGCGCCGGCAAGACTAGGGATAACAGGGTAATCGATTT | | |
| *UL105* G120V-rev | | CCAGCACCTGGATGCTGGAAGTCTTGCCGGCGCCCGCCGTGACGGTGACGAGCAGCGCGCGGAGCCAGTGTTACAACCAATTAACC | | |
| *UL105* G123S-for | | CTTCTTTCCCTTCCGCGCGCTGCTCGTCACCGGCACGGCGAGCGCCGGCAAGACTTCCAGCATTAGGGATAACAGGGTAATCGATTT | | |
| *UL105* G123S-rev | | GATTGGCCGCCAGCACCTGGATGCTGGAAGTCTTGCCGGCGCTCGCCGTGCCGGTGACGAGCAGCCAGTGTTACAACCAATTAACC | | |
| *UL105* G123V-for | | CTTCTTTCCCTTCCGCGCGCTGCTCGTCACCGGCACGGCGGTCGCCGGCAAGACTTCCAGCATTAGGGATAACAGGGTAATCGATTT | | |
| *UL105* G123V-rev | | GATTGGCCGCCAGCACCTGGATGCTGGAAGTCTTGCCGGCGACCGCCGTGCCGGTGACGAGCAGCCAGTGTTACAACCAATTAACC | | |
| *UL105* K126A-for | | CTTCCGCGCGCTGCTCGTCACCGGCACGGCGGGCGCCGGCGCGACTTCCAGCATCCAGGTGCTTAGGGATAACAGGGTAATCGATTT | | |
| *UL105* K126A-rev | | CGCAATCTAGATTGGCCGCCAGCACCTGGATGCTGGAAGTCGCGCCGGCGCCCGCCGTGCCGGGCCAGTGTTACAACCAATTAACC | | |
| *UL105* K126N-for | | CTTCCGCGCGCTGCTCGTCACCGGCACGGCGGGCGCCGGCAACACTTCCAGCATCCAGGTGCTTAGGGATAACAGGGTAATCGATTT | | |
| *UL105* K126N-rev | | CGCAATCTAGATTGGCCGCCAGCACCTGGATGCTGGAAGTGTTGCCGGCGCCCGCCGTGCCGGGCCAGTGTTACAACCAATTAACC | | |
| *UL105* T127A-for | | CCGCGCGCTGCTCGTCACCGGCACGGCGGGCGCCGGCAAGGCTTCCAGCATCCAGGTGCTGGCTAGGGATAACAGGGTAATCGATTT | | |
| *UL105* T127A-rev | | TCACGCAATCTAGATTGGCCGCCAGCACCTGGATGCTGGAAGCCTTGCCGGCGCCCGCCGTGCGCCAGTGTTACAACCAATTAACC | | |
| *UL105* D261E-for | | GGACCTCTCGGAGCTGTGCGAGAGCAATATCATCGTCATCGAGGAGTGCGGCCTTATGCTGCGTAGGGATAACAGGGTAATCGATTT | | |
| *UL105* D261E-rev | | CCACCACCTGCAGCATGTAGCGCAGCATAAGGCCGCACTCCTCGATGACGATGATATTGCTCTGCCAGTGTTACAACCAATTAACC | | |
| *UL105* D261N-for | | GGACCTCTCGGAGCTGTGCGAGAGCAATATCATCGTCATCAACGAGTGCGGCCTTATGCTGCGTAGGGATAACAGGGTAATCGATTT | | |
| *UL105* D261N-rev | | CCACCACCTGCAGCATGTAGCGCAGCATAAGGCCGCACTCGTTGATGACGATGATATTGCTCTGCCAGTGTTACAACCAATTAACC | | |
| *UL105* E262D-for | | CCTCTCGGAGCTGTGCGAGAGCAATATCATCGTCATCGACGACTGCGGCCTTATGCTGCGCTATAGGGATAACAGGGTAATCGATTT | | |
| *UL105* E262D-rev | | ACACCACCACCTGCAGCATGTAGCGCAGCATAAGGCCGCAGTCGTCGATGACGATGATATTGCGCCAGTGTTACAACCAATTAACC | | |
| *UL105* E262Q-for | | CCTCTCGGAGCTGTGCGAGAGCAATATCATCGTCATCGACCAGTGCGGCCTTATGCTGCGCTATAGGGATAACAGGGTAATCGATTT | | |
| *UL105* E262Q-rev | | ACACCACCACCTGCAGCATGTAGCGCAGCATAAGGCCGCACTGGTCGATGACGATGATATTGCGCCAGTGTTACAACCAATTAACC | | |
| *UL105* Q306A-for | | ACGCCGCGTGCCCTGCATCATCTGCGTCGGTTCGCCCACGGCGACCGAGGCGCTGGAGAGCCGTAGGGATAACAGGGTAATCGATTT | | |
| *UL105* Q306A-rev | | TTTGCGTGTAGTGGTCGTAGCGGCTCTCCAGCGCCTCGGTCGCCGTGGGCGAACCGACGCAGAGCCAGTGTTACAACCAATTAACC | | |
| *UL105* H82K-for | | CCCCTCTGCCGCAACTACCGCCGCGGCAACGACGACCGTTAAATCCTCCGCCTCCTCTTCTGCTAGGGATAACAGGGTAATCGATTT | |  |
| *UL105* H82K-rev | | CGGACGAAGCGGCAGCGGCGGCAGAAGAGGAGGCGGAGGATTTAACGGTCGTCGTTGCCGCGGGCCAGTGTTACAACCAATTAACC | |  |
| *UL105* H82R-for | | CCCCTCTGCCGCAACTACCGCCGCGGCAACGACGACCGTTCGCTCCTCCGCCTCCTCTTCTGCTAGGGATAACAGGGTAATCGATTT | |  |
| *UL105* H82R-rev | | CGGACGAAGCGGCAGCGGCGGCAGAAGAGGAGGCGGAGGAGCGAACGGTCGTCGTTGCCGCGGGCCAGTGTTACAACCAATTAACC | |  |
| *UL70* C881S-for  *UL70* C881S-rev | | CGTCAAGCGGCGCGACGGCGCGCGCACGCGGGATTTTCGCTCTCTCAACTACACGCACCGCAATAGGGATAACAGGGTAATCGATTT  CTTGCACGGTCTCTTGCGGGTTGCGGTGCGTGTAGTTGAGAGAGCGAAAATCCCGCGTGCGCG GCCAGTGTTACAACCAATTAACC | |  |
| *UL70* H886A-for | CGGCGCGCGCACGCGGGATTTTCGCTGTCTCAACTACACGGCCCGCAACCCGCAAGAGACCGTTAGGGATAACAGGGTAATCGATTT | |  |  |
| *UL70* H886A-rev | GCAGGTCGATGAACACTTGCACGGTCTCTTGCGGGTTGCGGGCCGTGTAGTTGAGACAGCGAAGCCAGTGTTACAACCAATTAACC- | |  |  |
| *UL70* C915S-for | CGAGCACAGCTACGCGCTCTGGGCCAGCCTCTGGAGCCGCTCTTTCACCAAAAAGTGTCACTCTAGGGATAACAGGGTAATCGATTT | |  |  |
| *UL70* C915S-rev | TGTGGACGTTTTTGGCGTTGGAGTGACACTTTTTGGTGAAAGAGCGGCTCCAGAGGCTGGCCC GCCAGTGTTACAACCAATTAACC | |  |  |
| *UL70* C920S-for | GCTCTGGGCCAGCCTCTGGAGCCGCTGTTTCACCAAAAAGTCTCACTCCAACGCCAAAAACGTTAGGGATAACAGGGTAATCGATTT | |  |  |
| *UL70* C920S-rev | TGATTTTGATGGAAATGTGGACGTTTTTGGCGTTGGAGTGAGACTTTTTGGTGAAACAGCGGCGCCAGTGTTACAACCAATTAACC | |  |  |

| Primers | Primer sequence |
| --- | --- |
| *UL105* - Externe 1 | AATCTCCTCGTGCAGGTGTG |
| *UL105* - Externe 2 | TTTCTCTTGGTCGAACCCGG |
| *UL105* - Interne 1 | CGCACCAATCCACGTAGAGA |
| *UL105* - Interne 2 | GATGTGTTCCCTGACGGTGT |
| *UL105* - 2 | CTCCTCCGCCTCCTCTTCT |
| *UL105* - 3 | AGAAGAGGAGGCGGAGGAG |
| *UL105* - 4 | GCTACGACCACTACACGCAA |
| *UL105* - 5 | TTCTGAATCAGCGCCGAGAG |
| *UL105* - 6 | ACTTGGCGCGCATCATTAAC |
| *UL105* - 7 | CATCGTCCTGGAAAAACGCC |
| *UL105* - 8 | TCGACGCGGATTCTTCTCAG |
| *UL105* - 9 | CTGAGAAGAATCCGCGTCGA |
| *UL105* - 10 | GTTAACGTTTCGCGCTTCGT |
| *UL105* - 11 | GACGAAGCGCGAAACGTTAA |
| *UL70* - Externe 1 | TTCTCGCAGTCCATGTCCAC |
| *UL70* - Externe 2 | GGACAACTGGGAACTCGGC |
| *UL70* - Interne 1 | CCTCCGAGAGATAGCCCTTG |
| *UL70* - Interne 2 | TCTTCCGCGAGATGGTGTTC |
| *UL70* - 2 | GCGAATTCAAGCACCTGGTG |
| *UL70* - 3 | CACCAGGTGCTTGAATTCGC |
| *UL70* - 4 | AATACTTCTCGCTCGACAACT |
| *UL70* - 5 | AGTTGTCGAGCGAGAAGTATT |
| *UL70* - 6 | CTTCAACGAACGTCTGCCC |
| *UL70* - 7 | AGCCGTAAATCAAAGTCGGC |
| *UL70* - 8 | ATGAACAGCGGCAACAGAC |
| *UL70* - 9 | ACGTCAGCGAGCAGTTTACC |
| *UL70* - 10 | TCGGGCGGTCTGATTTTGA |

**Supplementary Table 4: Primers used for screening and sequencing**.
